# A prognostic model for early risk stratification in adult community-acquired suspected CNS infections: multicenter development and external validation

**DOI:** 10.1186/s12879-025-11776-8

**Published:** 2025-11-17

**Authors:** Fei Tian, Zhaoyang Liu, Weibi Chen, Lili Cui, Dawei Shan, Huimin Zhang, Shuting Chai, Gang Liu, Linlin Fan, Guofeng Li, Le Yang, Jiatang Zhang, Jiahua Zhao, Fengqin Hou, Jianxin Du, Xinyu Huan, Ying Lv, Xun Huang, Rongrong Zhang, Liyong Wu, Yingfeng Wu, Yan Zhang

**Affiliations:** 1 Department of Neurology, Xuanwu Hospital, Capital Medical University, No. 45 Changchun Street, Xicheng District, Beijing, 100053 China; 2 Department of Emergency, Guizhou Provincial People’s Hospital, Guiyang, China; 3 Department of Neurology, The First Medical Center of the Chinese PLA General Hospital, Beijing, China; 4 Department of Infectious Diseases, Peking University First Hospital, Beijing, China; 5 Department of Neurosurgery, You’anmen Hospital, Fengtai District, Beijing, China; 6 Department of Neurology and Psychiatry, Shijitan Hospital, Capital Medical University, Beijing, China; 7 Department of Infectious Diseases, Xiangya Hospital, Central South University, Changsha, China; 8 Department of Intensive Care Medicine, The First Affiliated Hospital, University of Science and Technology of China, Hefei, China; 9 Department of Vascular Surgery, Xuanwu Hospital, Capital Medical University, No. 45 Changchun Street, Xicheng District, Beijing, 100053 China

**Keywords:** Central nervous system infections, Prognostic model, Risk stratification, Outcome prediction, Community-acquired infections, External validation

## Abstract

**Objective:**

Community-acquired central nervous system (CNS) infections remain a major cause of morbidity and mortality, particularly in resource-limited settings. Early prognostication is critical but challenged by delays in definitive diagnosis. This study aimed to develop and externally validate a simple prognostic model to support early risk stratification and intervention.

**Methods:**

We conducted a prospective multicenter cohort study in China (NCT04722328), enrolling 1,060 adults with suspected CNS infections. Patients from four hospitals were included in the training group (*n* = 742) for model development, while patients from three independent hospitals formed the external validation group (*n* = 318). Independent predictors were identified using least absolute shrinkage and selection operator (LASSO) and multivariable logistic regression. A nomogram was constructed to estimate individualized risk. Model performance was assessed via area under the receiver operating characteristic curve (AUC), concordance index (C-index), calibration plots, decision curve analysis (DCA), and clinical impact curves (CICs).

**Results:**

Six predictors were independently associated with poor outcomes: absence of headache, altered consciousness, respiratory failure, hypoproteinemia, low hemoglobin, and hyperglycemia. The model demonstrated strong discrimination in the training group (AUC 0.811; 95% CI 0.774–0.849) and excellent calibration, with DCA indicating clear clinical benefit. External validation confirmed robust performance (AUC 0.855; 95% CI 0.800–0.910), supporting the model’s generalizability.

**Conclusion:**

We established a prognostic model to support early identification of severe cases and guide timely comprehensive management in adult patients with suspected community-acquired CNS infections.

**Clinical registration:**

ClinicalTrials.gov (NCT04722328).

## Highlights

1. Developed and externally validated a prognostic model for adverse outcomes in adult community-acquired suspected meningitis and encephalitis using a multicenter prospective cohort.

2. Identified six routinely available clinical and laboratory predictors independently associated with poor prognosis.

3. Constructed a clinically applicable nomogram to facilitate early individualized risk stratification and support timely management decisions.

## Introduction

Community-acquired central nervous system (CNS) infections, including meningitis and encephalitis, are neurological emergencies and remain a major cause of mortality and long-term disability in both high-income and low-income countries [1]. Although therapeutic advances have been made, the global burden of CNS infections persists, especially in resource-limited regions where pathogen diversity, delayed access to care, and diagnostic challenges contribute to poor clinical outcomes [2, 3]. Early identification of patients at high risk for unfavorable outcomes is crucial for timely intervention; however, reliable and widely applicable prognostic tools are still lacking.

Several studies have explored clinical predictors of adverse outcomes in patients with bacterial or viral CNS infections. However, many existing prognostic models are limited by retrospective, single-center designs and a narrow focus on specific pathogens, such as *Streptococcus pneumoniae* or herpesviruses [4, 5]. Moreover, a substantial proportion of patients empirically treated for suspected CNS infections lack microbiological confirmation yet still face a considerable risk of poor outcomes — a persistent clinical challenge that remains difficult to overcome in the near term [6]. Nevertheless, these patients are often underrepresented in model development and validation efforts. In addition, existing models frequently perform suboptimally when externally validated across heterogeneous populations, and their generalizability remains uncertain, particularly in resource-limited or diagnostically constrained settings [7]. These limitations highlight the urgent need for robust and generalizable tools that can accommodate diverse clinical presentations and enable early risk stratification, even in the absence of a confirmed etiological diagnosis.

Recognizing these limitations, we conducted a prospective, multicenter cohort study in China to develop and externally validate a prognostic model for adverse outcomes in adult patients with community-acquired suspected CNS infections. Our model integrates a broad set of routinely available clinical and laboratory parameters into a nomogram—a graphical, user-friendly tool designed to facilitate individualized risk assessment. With a focus on simplicity and clinical applicability, the model aims to support early therapeutic decision-making, improve triage accuracy, and optimize resource utilization, particularly in settings where diagnostic confirmation is frequently delayed or unavailable.

## Methods

### Study design and population

The study cohort was derived through the Prevention and Control of Neurological Infection (PACNI) Program, which was conducted in China and registered on ClinicalTrials.gov (NCT04722328) with a registration date of January 19, 2021. This prospective observational study included seven multicenter sites and used the Comprehensive Clinical Infection Database (IDCI) system, available at https://idci.capitalbio.com. The IDCI system is part of a major research initiative funded by the Ministry of Science and Technology of China and was used within the Chinese Adult Encephalitis Trials (CAET) dataset. All procedures complied with applicable legal and institutional guidelines and received approval from the appropriate institutional review committees. Ethical approval was obtained from the Ethics Committee of Xuanwu Hospital, Capital Medical University (Approval No: Xuanwu [2020]104), with additional approvals from local review boards at each participating site. Informed consent was obtained for all human subject experimentation.

The study was conducted between March 2021 and September 2022, enrolling a total of 1,612 patients who were hospitalized with clinically suspected meningitis or encephalitis across seven tertiary teaching hospitals in China. Patients were divided into two groups: 70% (from four tertiary hospitals) were included in the training group for model development and nomogram construction, while the remaining 30% (from three independent tertiary hospitals) formed the external validation group. This centre-based stratification ensured both robust model derivation and reliable external validation, thereby enhancing the generalisability and clinical applicability of the prognostic tool.

### Inclusion criteria

1. Participants aged ≥ 18 years;
2. Symptoms must have occurred within the past month;
3. A pre-onset modified Rankin Scale (mRS) score of less than 2 [8];
4. Clinical suspicion of meningitis or encephalitis (M/E) based on:

①For meningitis: Symptoms such as fever, headache, vomiting, and nuchal rigidity, combined with cerebrospinal fluid (CSF) pleocytosis (≥ 5 white cells/µL) or evidence of meningeal enhancement on neuroimaging [9].

 ②For encephalitis: Altered mental status persisting for more than 24 h without an identifiable cause, along with at least two of the following: fever (≥ 38 °C) or a history of fever during the current illness, seizures or focal neurological signs, CSF pleocytosis (>5/mm^3^), an electroencephalogram (EEG) suggestive of encephalitis (generalized slow waves, recurrent spikes or spike-and-slow-wave complexes predominantly in the temporal regions, or lateralized periodic epileptiform discharges [LPDs]), or neuroimaging findings consistent with encephalitis [10];

 (5) Informed consent obtained from all participants or their designated legal representatives.

An mRS score < 2 was selected to ensure that poor outcomes could be attributed more confidently to the CNS infectionsitself rather than pre-existing disability [11, 12].

### Exclusion criteria

Patients were excluded if they met any of the following criteria:

(1) Incomplete CSF examination;

(2) Encephalopathy resulting from trauma, metabolic disorders, neoplasia, alcohol abuse, or other non-infectious causes;

(3) Diagnosed with autoimmune encephalitis during the clinical course;

(4) Pre-existing severe multi-organ failure;

(5) Major surgical intervention within the past six months;

(6) Presence of malignant neoplasm.

### Data collection

The CAET database captures comprehensive demographic data, including gender and age, as well as detailed information on symptom onset and medical or personal history, covering conditions like diabetes, smoking, and drinking. It also includes precise documentation of potential triggers for CNS infections, such as prodromal symptoms like fever or respiratory issues. The database records a wide range of symptoms, including headache, vomiting, seizures, respiratory failure, and mental manifestations. Additionally, it encompasses data from auxiliary tests, such as lumbar punctures, blood cell counts, hemoglobin levels, plasma glucose, and hypoalbuminemia. This extensive dataset facilitates in-depth analysis for the study of CNS infections.

### Clinical procedures

(1) Hematological testing: Within 72 h of enrollment, patients underwent comprehensive hematological assessments, including complete blood count, full biochemical panel, procalcitonin, C-reactive protein, interleukin-6, lymphocyte subset typing, and immunoglobulin levels. Blood cultures were obtained in patients with a body temperature exceeding 38 °C.

(2) Lumbar puncture: Lumbar puncture was performed within seven days of enrollment to measure CSF opening pressure and collect CSF samples for routine analysis, cytology, biochemistry, and microbiological testing.

(3) Microbiological methods included:

① For bacteria: Gram staining and culture;

② For viruses: Nucleic acid amplification testing or CSF antibody testing;

③ For Mycobacterium tuberculosis: Acid-fast staining, culture, tuberculosis-specific T cell enzyme-linked immunospot assay (TB-spot), and automated real-time fluorescent quantitative PCR for Mycobacterium tuberculosis nucleic acid and rifampicin resistance (Xpert MTB/RIF);

④ For Cryptococcus or Cryptococcus gattii: Cryptococcal antigen testing or fungal culture.

⑤ Metagenomic next-generation sequencing (mNGS) of CSF was performed in patients where conditions permitted.

(4) Anti-infective treatment: Empirical antimicrobial therapy was initiated based on clinical presentation. Once a definitive pathogen was identified through CSF analysis, targeted therapy was administered. If CSF pathogen detection was negative or inconsistent with clinical findings, therapy was adjusted according to CSF characteristics indicative of viral, bacterial, tuberculous, or fungal infections. Pathogen-directed treatment was re-evaluated after one week based on clinical response.

### Outcome evaluation

The prognostic assessment was performed six months after treatment using mRS. The mRS ranges from 0 to 6, with higher scores reflecting worse outcomes. An mRS score greater than 2 denotes a poor outcome, while a score of 2 or lower indicates a favorable outcome.

### Statistical analysis

Statistical analyses were conducted in R (version 4.3.2) using haven, caTools, compareGroups, glmnet, autoReg, rms, pROC, ResourceSelection, rmda, ggDCA, and ggplot2. Statistical significance was defined as a two-sided p-value < 0.05.

All eligible cases from the training group were analyzed descriptively. Continuous variables were presented as medians and interquartile ranges (IQRs) and compared using the Mann–Whitney U test based on non-normal distribution assessed by the Kolmogorov-Smirnov test, Shapiro-Wilk test, and Q-Q plots. Categorical variables were expressed as counts and percentages and compared using the chi-square test or Fisher’s exact test, as appropriate.

Model development began with least absolute shrinkage and selection operator (LASSO) regression to screen potential predictors, followed by multivariable logistic regression to identify independent predictors. Variables with significant associations in univariate analyses were entered into the multivariable model. A nomogram was constructed based on the final multivariable model.

Model performance was evaluated using the area under the receiver operating characteristic curve (AUC), concordance index (C-index), calibration plots, and the Hosmer-Lemeshow goodness-of-fit test. Clinical utility was assessed through decision curve analysis (DCA) and clinical impact curves (CICs).

External validation was performed in an independent cohort using the same statistical procedures.

### Role of the funding source

The funder of the study had no role in study design, data collection, data analyses, data interpretation, or writing of the report.

## Results

**Fig. 1:**
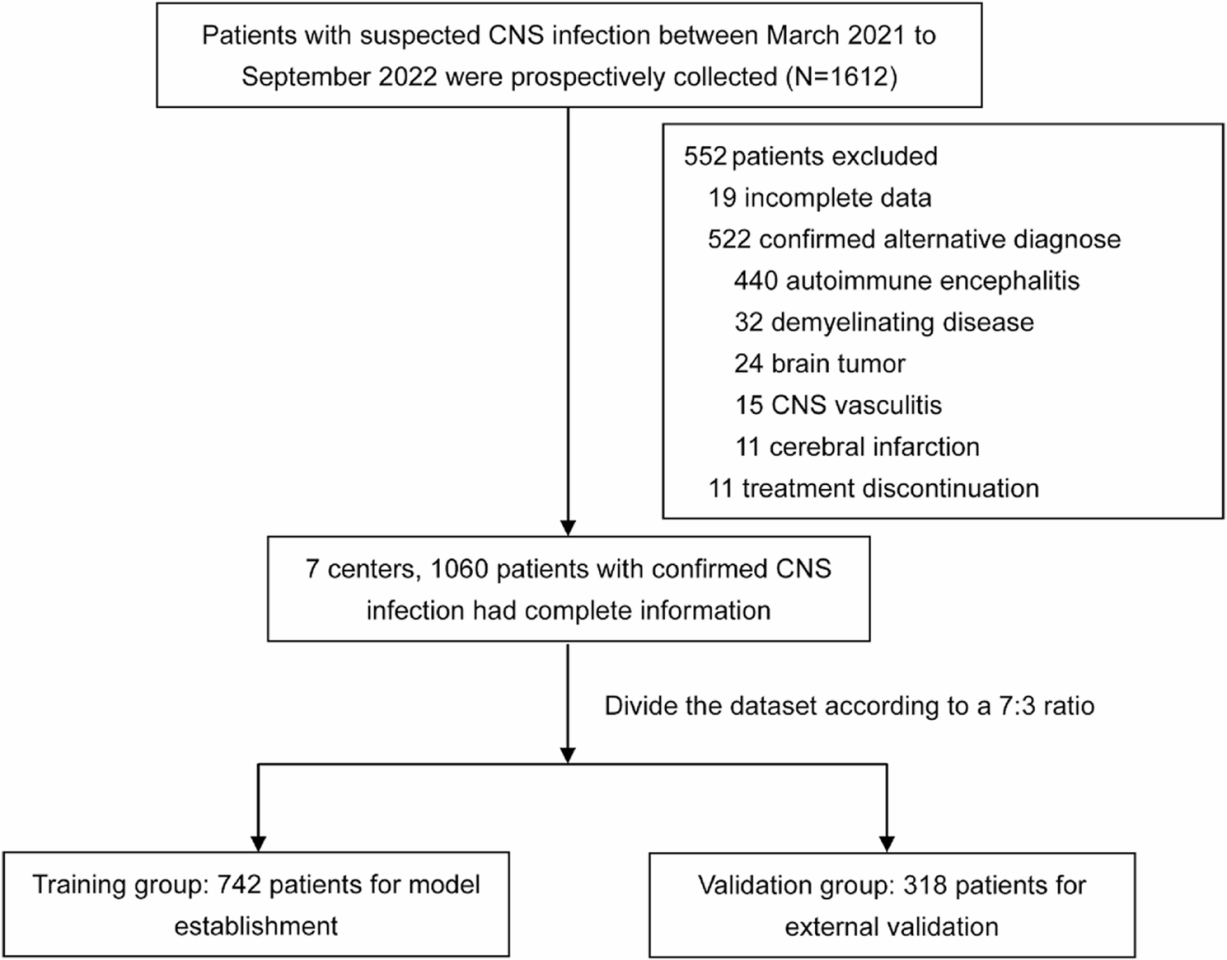
Flow chart of patient selection and dataset allocation. CNS: central nervous system

Of the 1612 patients initially suspected of having CNS infections, 552 were excluded due to incomplete data (*n* = 19), confirmed alternative diagnoses (*n* = 522), or treatment discontinuation (*n* = 11), resulting in 1060 patients included for analysis. These were subsequently divided into a training group (*n* = 742) for model development and a validation group (*n* = 318) for external validation of the nomogram (Fig. 1).

### Pathogen composition

The etiological distribution of the 1,060 patients with CNS infections is summarized in Table 1. Viral infections were the most common, followed by non-tuberculous bacterial infections, tuberculosis-related infections, fungal infections, and syphilis. Importantly, across all pathogen categories, a substantial proportion of patients were clinically diagnosed without microbiological confirmation, underscoring the ongoing challenge of achieving definitive etiological diagnoses in CNS infections.

**Table 1:** Pathogen distribution in adult patients with suspected CNS infections

Pathogen Category	Cases	% of total patients (*n* = 1,060)	Specific Pathogen	Number (*n*)	Percentage within Category (%)
Virus	661	62.4	HSV-1 HSV-2 VZV EBV CMV HHV-6 HHV-7 HHV-8 Polyomavirus Parvovirus Enterovirus JEV Clinically suspected viral infection*	165 63 11 76 25 6 7 7 1 1 27 11 261	25.0 9.5 1.7 11.5 3.8 0.9 1.1 1.1 0.2 0.2 4.1 1.7 ***39.5***
Bacteria	209	19.7	Listeria Pseudomonas aeruginosa Streptococcus Klebsiella pneumoniae Staphylococcus Brucella Micromonospora Clinically suspected bacterial infection**	8 8 38 3 6 38 2 106	3.8 3.8 18.2 1.4 2.9 18.2 1.0 ***50.7***
Tuberculosis	111	10.5	Tuberculosis Clinically suspected TB infection**	59 52	53.2 ***46.8***
Fungus	69	6.5	Cryptococcus Candida albicans Clinically suspected fungal infection*	20 14 34	29.0 20.3 ***49.3***
Syphilis	10	0.9	Syphilis	10	100

*HSV* Herpes simplex virus, *VZV* Varicella-zoster virus, *EBV* Epstein–Barr virus, *CMV* Cytomegalovirus, *HHV* Human herpesvirus, *JEV* Japanese encephalitis virus, *TB* Tuberculosis, *CSF* Cerebrospinal fluid

*Clinically suspected infections refer to cases without definitive microbiological confirmation but diagnosed based on clinical features, imaging findings, cerebrospinal fluid (CSF) analysis, and response to anti-infective treatment

### Baseline characteristics of the training and validation groups

In the training group, the median age of patients was 46 years (interquartile range [IQR]: 31–59 years), and 35.2% were female. Based on medical history, 12.0% had diabetes, 30.9% were smokers, and 23.7% reported a history of alcohol consumption. Among patients with suspected CNS infections 63.2% had viral infections, 18.6% had non-tuberculous bacterial infections, 11.2% had tuberculosis-related infections, 5.9% had fungal infections, and 1.1% had syphilitic infections.

The demographic and clinical characteristics of the external validation group were comparable to those of the training group. The median age was 46 years (IQR: 32–58 years), and 34.9% were female. The prevalence of diabetes was 11.9%, with 31.4% of patients being smokers and 18.9% reporting a history of alcohol consumption. Regarding the distribution of suspected CNS infections, 61.0% had viral infections, 23.3% had non-tuberculous bacterial infections, 8.8% had tuberculosis-related infections, 7.9% had fungal infections, and 0.6% had syphilitic infections.

Statistical comparisons revealed no significant differences between the training and external validation groups across the variables examined, supporting the comparability of the two groups (Table 2).

**Table 2:** Baseline Characteristics of the Training and Validation Groups

Demographics	**All eligible patients (n = 1060)**		*p*** value**
	Training group(n = 742)	Validation group(n = 318)	
Age (years, Median and IQR)	46 [31, 59]	46 [32, 58]	0.897
Sex (n, %)			
Female	261 (35.2)	111 (34.9)	0.989
Male	481 (64.8)	207 (65.1)	
Medical and personal history (n, %)			
Diabetes	89 (12.0)	38 (11.9)	1.000
Smoking	229 (30.9)	100 (31.4)	0.908
Drinking	176 (23.7)	60 (18.9)	0.097
CNS infectionstype (n, %)			
Virus	469 (63.2)	192 (60.4)	0.542
Bacteria	138 (18.6)	71 (22.3)	0.097
Tuberculosis	83 (11.2)	28 (8.8)	0.293
Fungus	44 (5.9)	25 (7.9)	0.302
Syphilis	8 (1.1)	2 (0.6)	0.116
Symptoms at Presentation (n, %)			
Fever	509 (68.6)	226 (71.1)	0.467
Respiratory infection	101 (13.6)	55 (17.3)	0.145
Diarrhea	22 (3.0)	9 (2.8)	1.000
Headache	424 (57.1)	191 (60.1)	0.415
Vomiting	222 (29.9)	107 (33.6)	0.258
Stiff neck	193 (26.0)	77 (24.2)	0.590
Mental symptoms	346 (46.6)	144 (45.3)	0.737
Seizure	170 (22.9)	70 (22.0)	0.810
Status Epilepticus	43 (5.8)	14 (4.4)	0.440
Altered consciousness	287 (38.7)	115 (36.2)	0.481
Respiratory failure	58 (7.8)	28 (8.8)	0.676
Laboratory Examinations			
Neutrophil (10^9^/L, Median and IQR)	4.84 [3.22, 7.24]	4.79 [3.30, 7.29]	0.805
Neutrophil% (Median and IQR)	70.8 [60.4, 81.4]	71.8 [61.2, 80.9]	0.765
Lymphocyte (10^9^/L, Median and IQR)	1.31 [0.84, 1.81]	1.35 [0.87, 1.86]	0.366
Lymphocyte% (Median and IQR)	20.0 [11.3, 29.1]	19.6 [11.4, 28.9]	0.767
Hemoglobin (g/L, Median and IQR)	131 [117, 143]	131 [117, 143]	0.958
Hypoalbuminemia (n, %)	217 (29.2)	90 (28.3)	0.813
Blood glucose (mmol/L, Median and IQR)	5.55 [4.76, 7.12]	5.57 [4.82, 7.00]	0.781
Cerebrospinal Fluid Pressure (n, %)			
Low	16 (2.2)	3 (0.9)	0.306
Normal	356 (48.0)	162 (50.9)	
High	370 (49.9)	153 (48.1)	
Functional Outcome (mRS) (n, %)			
Good outcome (mRS 0–2)	565 (76.1)	244 (76.7)	0.900
Poor outcome (mRS 3–6)	177 (23.9)	74 (23.3)	

Data are presented as n (%) for categorical variables; median [interquartile range, IQR] for continuous variables

*CNS* Central nervous system, *IQR* Interquartile range, *mRS* modified Rankin Scale, Altered consciousness refers to any disturbance of consciousness level as assessed clinically, Hypoalbuminemia is defined as a serum albumin concentration of less than 35 g/L

### Variable selection and model refinement

**Fig. 2:**
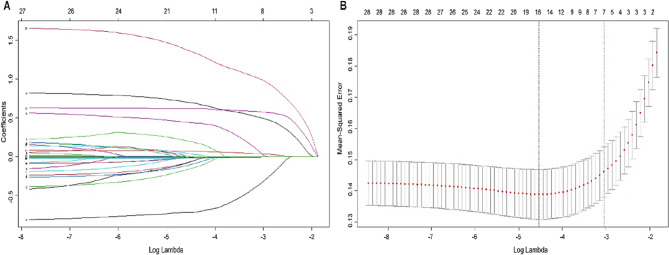
LASSO regression results. **A** Plot of regression coefficients versus log(λ); (**B**) Plot of MSE versus log(λ). λ.min represents the log(λ) value at which the MSE is minimized, whereas λ.1se represents the most parsimonious model whose error is within one standard error of the minimum MSE

To address multicollinearity and prevent overfitting in the high-dimensional dataset, LASSO regression was applied to the initial set of 28 variables within the training cohort. Using 100-fold cross-validation, the number of variables was first reduced to 16 at the value of log(λ) corresponding to the minimum mean squared error (MSE). Further refinement was performed at the log(λ) value corresponding to one standard error (SE) above the minimum MSE, resulting in the selection of 7 optimal variables: headache, mental symptoms, altered consciousness, respiratory failure, hypoalbuminemia, hemoglobin levels, and plasma glucose levels (Fig. 2).

Subsequent univariate and multivariable logistic regression analyses demonstrated that mental symptoms were not independently associated with poor outcomes (P >0.05; Table 3). Therefore, the final model incorporated the remaining six statistically significant variables for nomogram construction.

**Table 3:** Univariate and Multivariate Logistic Regression Analysis in the Training Group

Variables	**Good outcome(n = 565)**	**Poor outcome (n = 177)**	*p*^**a**^ **Value**	**OR (95% CI)**	*p*^b^** Value**	**OR (95% CI)**
Age (years, Median and IQR)	43 [30, 57]	52 [34, 65]	<0.001	1.02 (1.01–1.03)	0.329	0.99 (0.98–1.01)
Sex (n, %)						
Female	201 (35.6)	60 (33.9)	0.683	0.93 (0.65–1.33)	-	-
Male	364 (64.4)	117 (66.1)				
Medical and personal history (n, %)						
Diabetes	67 (11.9)	22 (12.4)	0.838	1.05 (0.63–1.76)	-	-
Smoking	170 (30.1)	59 (33.3)	0.415	1.16 (0.81–1.67)	-	-
Drinking	136 (24.1)	40 (22.6)	0.688	0.92 (0.62–1.38)	-	-
CNS infection type (n, %)						
Virus	359 (63.5)	110 (62.1)	0.737	0.94 (0.66–1.34)		
Bacteria	109 (19.3)	29 (16.4)	0.386	0.82 (0.52–1.29)	-	-
Tuberculosis	62 (11.0)	21 (11.9)	0.743	1.09 (0.65–1.85)	-	-
Fungus	29 (5.1)	15 (8.5)	0.104	11.71 (0.90–3.27)	-	-
Syphilis	6 (1.1)	2 (1.1)	1.000	0.94 (0.19–4.70)	-	-
Symptoms at Presentation (n, %)						
Fever	387 (68.5)	122 (68.9)	0.914	1.02 (0.71–1.47)	-	-
Respiratory infection	68 (12.0)	33 (18.6)	0.026	1.67 (1.06–2.64)	0.960	1.01 (0.57 – 1.79)
Diarrhea	15 (2.7)	7 (4.0)	0.377	1.51 (0.61–3.76)	-	-
Headache	351 (62.1)	73 (41.2)	<0.001	0.43 (0.30–0.60)	0.001	0.49 (0.32 – 0.75)
Vomiting	177 (31.3)	45 (25.4)	0.135	0.75 (0.51–1.10)	-	-
Stiff neck	143 (25.3)	50 (28.2)	0.437	1.16 (0.80–1.70)	-	-
Mental symptoms	267 (47.3)	79 (44.6)	0.541	0.90 (0.64–1.26)	-	-
Seizure	107 (18.9)	63 (35.6)	<0.001	2.37 (1.63–3.43)	0.858	1.05 (0.64 – 1.72)
Status Epilepticus	16 (2.8)	27 (15.3)	<0.001	6.18 (3.24–11.76)	0.520	1.34 (0.55 – 3.24)
Altered consciousness	164 (29.0)	123 (69.5)	<0.001	5.57 (3.85–8.05)	< 0.001	2.46 (1.57 – 3.84)
Respiratory failure	13 (2.3)	45 (25.4)	<0.001	14.48 (7.59–27.61)	< 0.001	5.04 (2.31 – 11.00)
Laboratory examinations						
Neutrophil (10^9^/L, Median and IQR)	4.40 [3.09, 6.62]	6.22 [4.03, 9.09]	<0.001	1.10 (1.05–1.14)	0.257	0.96 (0.89–1.03)
Neutrophil% (Median and IQR)	67.4 [58.8, 78.2]	79.2 [68.7, 86.4]	<0.001	1.05 (1.04–1.07)	0.742	1.01 (0.96–1.06)
Lymphocyte (10^9^/L, Median and IQR)	1.43 [1.00, 1.87]	0.91 [0.63, 1.56]	<0.001	0.42 (0.32–0.56)	0.945	0.99 (0.77–1.27)
Lymphocyte% (Median and IQR)	22.6 [13.6, 30.4]	12.1 [6.75, 22.1]	<0.001	0.94 (0.92–0.95)	0.566	0.98 (0.93–1.04)
Hemoglobin (g/L, Median and IQR)	133 [120, 145]	122 [105, 136]	<0.001	0.97 (0.96–0.98)	0.005	0.98 (0.97–1.00)
Hypoalbuminemia (n, %)	117 (20.7)	100 (56.5)	<0.001	4.97 (3.47–7.13)	0.002	2.08 (1.31–3.31)
Blood glucose (mmol/L, Median and IQR)	5.29 [4.69, 6.54]	6.77 [5.36, 9.68]	<0.001	1.19 (1.13–1.26)	0.004	1.10 (1.03–1.18)
Cerebrospinal Fluid Pressure (n, %)						
Low	11 (1.9)	5 (2.8)				
Normal	280 (49.6)	76 (42.9)	0.353	0.60 (0.20–1.77)	-	-
High	274 (48.5)	96 (54.2)	0.637	0.77 (0.26–2.28)	-	-

Data are presented as odds ratio (OR) with 95% confidence intervals (CI)

*IQR* Interquartile range, *OR* Odds ratio, *CI* Confidence interval

a: Test by univariate logistic regression analysis

b: Test by multivariate logistic regression analysis

### Development of the prediction nomogram

**Fig. 3:**
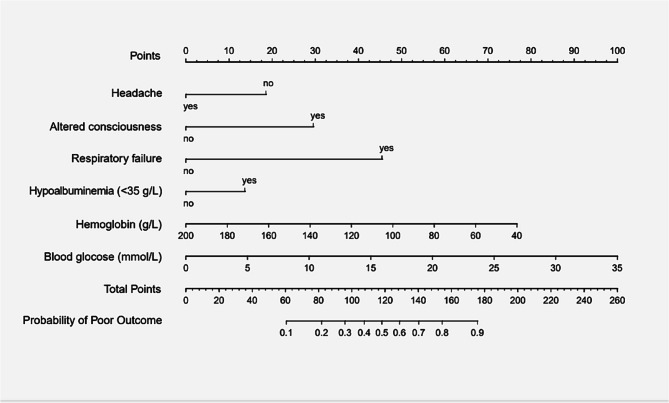
Nomogram for predicting poor outcomes in patients with CNS infections.To use the nomogram for estimating the risk of a poor outcome in an individual patient, the following steps are applied:(1) Identify the patient's value for each variable and locate the corresponding point on the respective axis;(2) Draw a vertical line upward from each variable axis to the Points scale at the top;(3) Sum the points assigned for all variables and locate the total score on the Total Points scale;(4) Draw a vertical line downward from the Total Points scale to the predicted probability axis to determine the patient's estimated risk of a poor outcome

A prediction nomogram was developed to facilitate individualized risk assessment of poor outcomes in patients with community-acquired CNS infections, based on the results of a multivariate logistic regression analysis (Fig. 3). The nomogram incorporates six variables identified as significant predictors, including altered consciousness, respiratory failure, hypoalbuminemia, reduced hemoglobin levels, and elevated blood glucose levels. Notably, the presence of headache was associated with a reduced risk of poor outcomes.

### Model performance and clinical utility

**Fig. 4:**
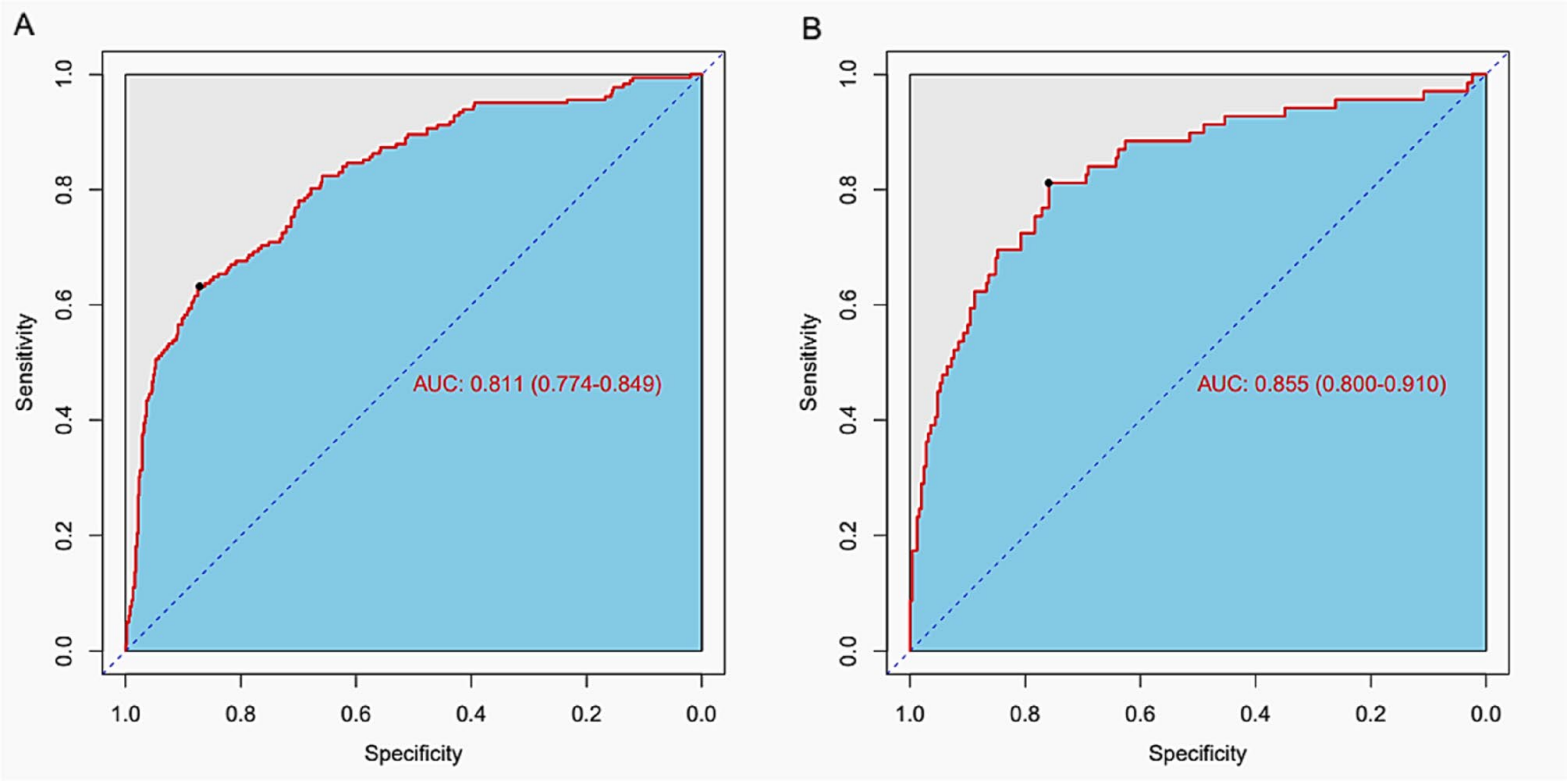
ROC curves for the risk of poor outcome. **A** Training group. **B** Validation group. ROC = Receiver Operating Characteristic curve; AUC = Area Under the ROC Curve

The model demonstrated good discrimination, with an AUC of 0.811 (95% CI: 0.774–0.849) in the training group and 0.855 (95% CI: 0.800–0.910) in the validation group (Fig. 4 A and 4B). The ROC curves showed a good balance between sensitivity and specificity, and the areas under the curve indicated that the model had a strong ability to distinguish between patients with and without poor prognosis in both the training and validation datasets.

**Fig. 5:**
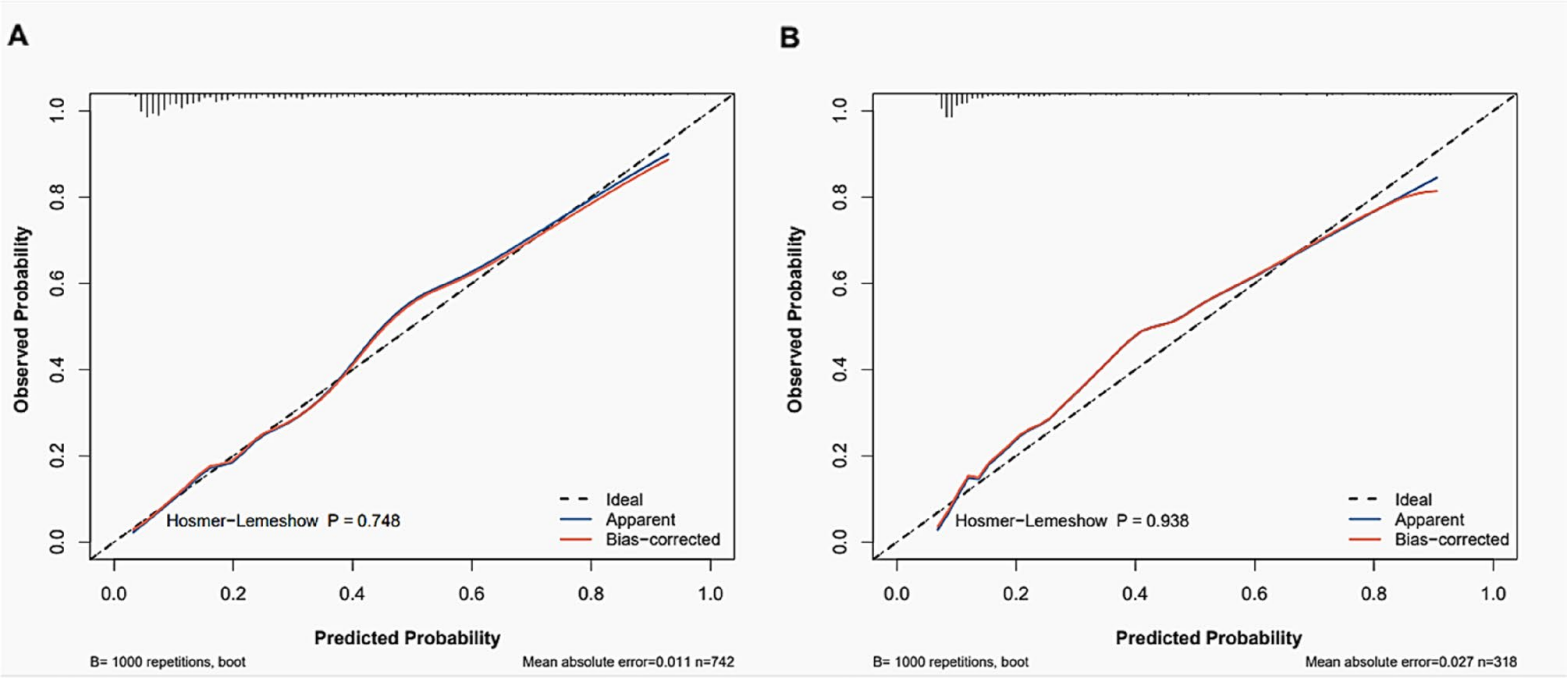
Calibration curves for poor outcome prediction. **A** Training group. **B** Validation group

The Hosmer-Lemeshow test was used to evaluate the calibration of the model, yielding p-values of 0.748 and 0.938 in the training and validation groups, respectively. These findings indicate a satisfactory agreement between the predicted probabilities and observed outcomes, as shown by the calibration curves (Fig. 5 A and 5B).

**Fig. 6:**
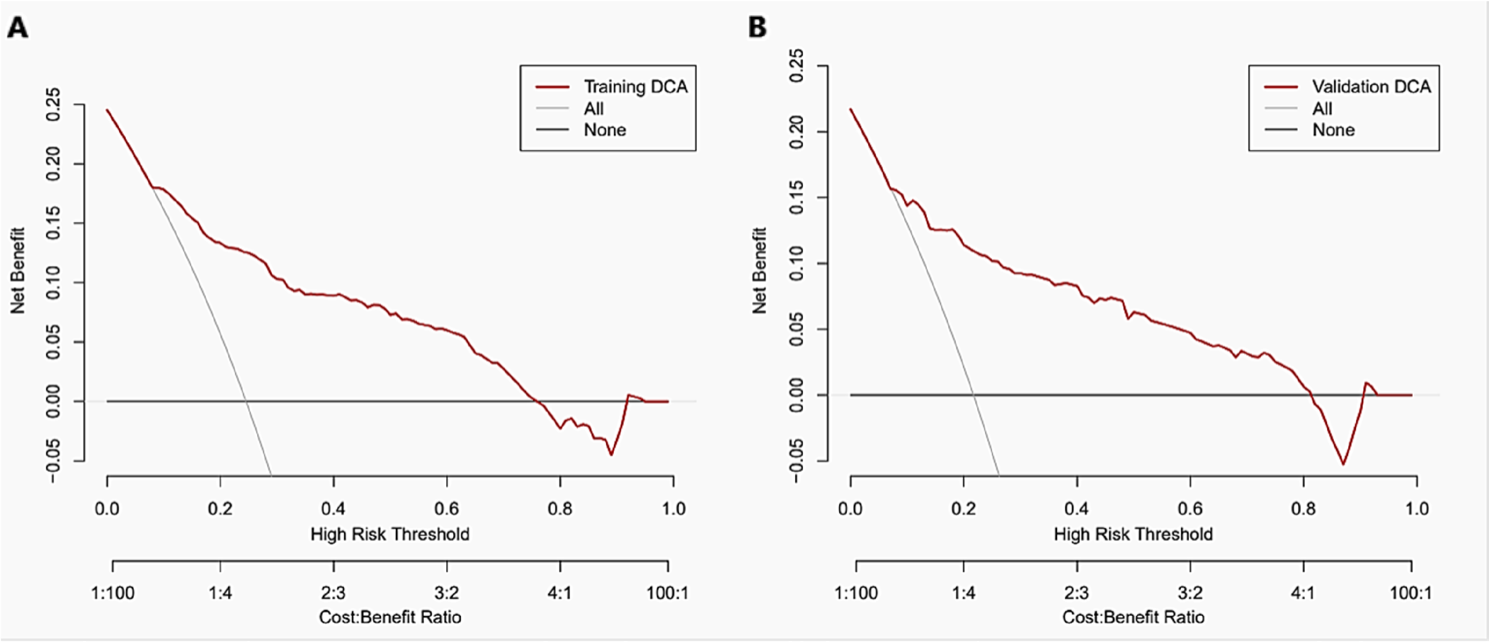
Decision curve analysis for poor outcome prediction. **A** Training group. **B** Validation group

DCA demonstrated a significant net benefit of the predictive model in both the training (Fig. 6 A) and validation groups (Fig. 6B).

**Fig. 7:**
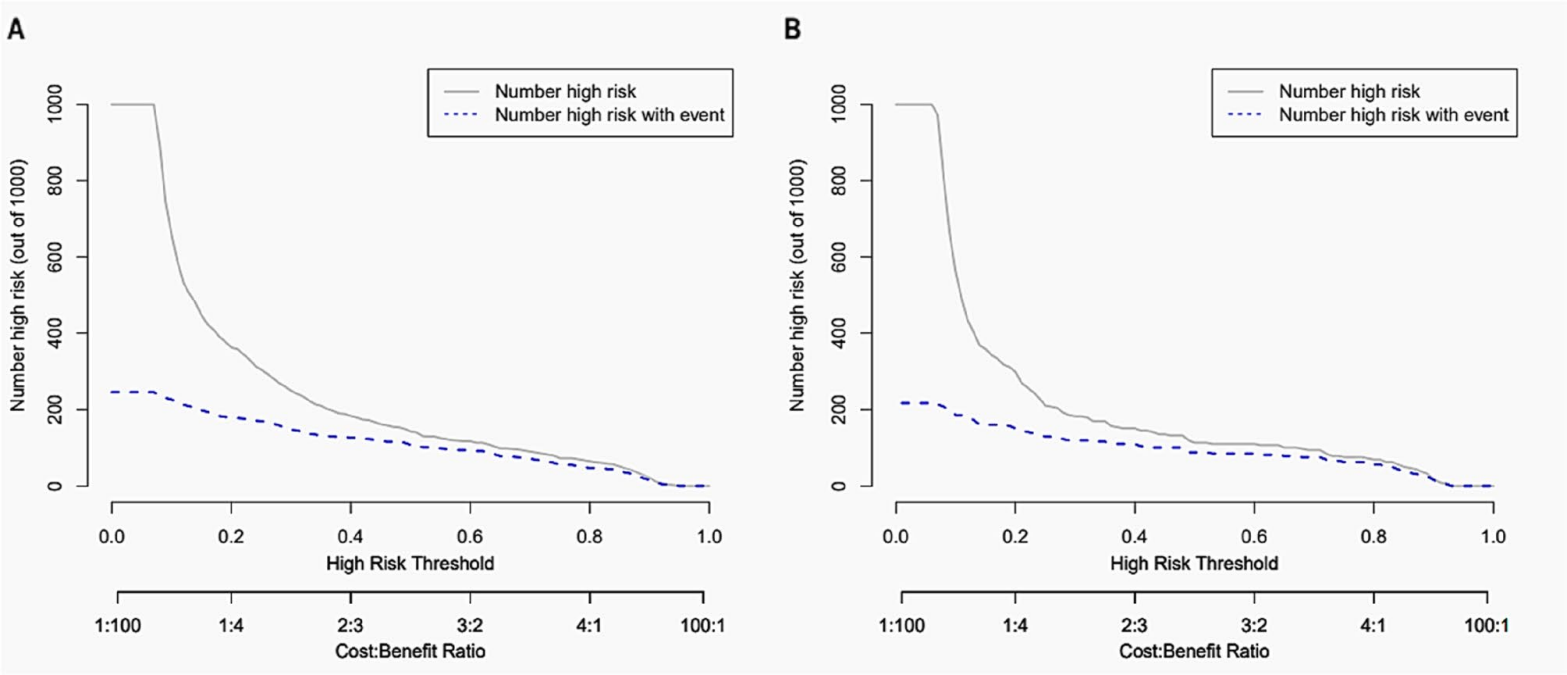
Clinical impact curves for poor outcome prediction. **A** Training group. **B** Validation group

Additionally, the CIC results indicate that the model demonstrates good discrimination (i.e., the ability to distinguish between high-risk and low-risk patients), applicability (generalizability across different scenarios or populations), and overall performance (predictive effectiveness) in both the training (Fig. 7 A) and validation groups (Fig. 7B).

## Discussion

In our multicenter, prospective study, we identified six significant predictors of outcomes in community-acquired suspected adult CNS infections. These included headache, which emerged as a protective factor, and impaired consciousness, respiratory failure, hypoalbuminemia, reduced hemoglobin, and elevated plasma glucose, all of which were associated with poor outcomes. Using these predictors, we developed a nomogram capable of accurately forecasting unfavorable outcomes within six months post-treatment, offering a practical tool for early risk stratification and clinical decision-making.

In our analysis, the occurrence of headaches in patients with CNS infections correlated with more favorable outcomes. This association can be explained by several indirect factors: (1) Early symptom recognition, such as the presence of headaches, can expedite medical intervention, leading to prompt diagnosis and treatment[13, 14]. (2) Headaches in the context of CNS infections may indicate an active immune response, which plays a crucial role in controlling the infection. Pro-inflammatory cytokines, including interleukin 1, tumor necrosis factor alpha, and interleukin 6, are known to activate brain nerve endings, resulting in the pain experienced as headaches. Additionally, the interaction between the nervous and immune systems, particularly involving cells like microglia and astrocytes, triggers the release of inflammatory mediators that contribute to headache symptoms[15, 16].

Impairment of consciousness ensues from damage to the brainstem reticular activating system, bilateral thalamus, and extensive cerebral hemispheres[17]. These disturbances often reflect severe underlying pathologies, such as inflammation, cerebral edema, and increased intracranial pressure[18, 19]. When intracranial pressure exceeds the brain's compensatory capacity, cerebral perfusion pressure declines, leading to tissue hypoxia and impaired glucose delivery. This results in metabolic dysfunction, energy failure, and ultimately neuronal death and global cerebral dysfunction. Such pathophysiological cascades are closely linked to poor clinical outcomes and increased mortality rates[20–22]. Consistent with previous findings, our study confirms a strong association between alterations in consciousness and adverse prognoses in patients with CNS infections.

Our findings highlight the significant role of respiratory failure in determining poor outcomes in patients with CNS infections. The mechanisms underlying respiratory failure in these cases are complex. Infection can directly compromise the respiratory centers in the brainstem, resulting in hypoventilation and respiratory insufficiency[23, 24]. Hypoventilation leads to hypoxemia, and hypoxia, in turn, causes neurological deterioration and irreversible neuronal injury through multiple mechanisms, including energy metabolism disruption, oxidative stress, inflammatory responses, apoptosis, and blood-brain barrier dysfunction[25]. Systemic inflammation, a hallmark of severe infections, may precipitate acute respiratory distress syndrome (ARDS), further impairing pulmonary function[26, 27]. Additionally, neurological deficits like impaired consciousness or cranial nerve palsies can elevate the risk of aspiration pneumonia, a prevalent and severe complication in this patient group[28, 29]. These factors collectively contribute to the adverse outcomes observed in our study.

Hypoalbuminemia, frequently observed in critically ill patients, is typically associated with a poor prognosis, especially in the context of infection and systemic inflammation. While direct evidence on the impact of hypoalbuminemia on CNS infections is limited, related research provides valuable insights. Firstly, The compromised nutritional status associated with low serum albumin levels impairs immune function, reducing the body's ability to combat infections effectively[30, 31]. Secondly, hypoalbuminemia is linked to altered fluid distribution due to decreased oncotic pressure, leading to edema and potentially exacerbating organ dysfunction by impairing perfusion and oxygen delivery[32]. This fluid imbalance can significantly affect the prognosis of critically ill patients by contributing to respiratory failure, delayed wound healing, and increased susceptibility to infection[33]. The inflammatory response characteristic of critical illness further complicates the scenario. This inflammation-induced hypoalbuminemia not only signals severe disease but also contributes to a vicious cycle of worsening inflammation, infection risk, and overall morbidity[34].

Similar to the hypoxemia resulting from respiratory failure, hemoglobin levels play a pivotal role in determining outcomes in adult CNS infections by ensuring adequate oxygen transport and tissue perfusion[35, 36]. Sufficient oxygen delivery is essential for maintaining neuronal function and supporting an effective immune response[37, 38]. Anemia can impair oxygenation, exacerbate neuronal injury, and compromise host defense mechanisms, thereby worsening infection outcomes[39–41]. Maintaining optimal hemoglobin levels may help preserve tissue oxygenation, promote infection control, and reduce neurological damage[42].

Hyperglycemia has consistently been associated with poor prognoses in patients, making it a key adverse prognostic factor in our model. It exacerbates CNS damage by intensifying the inflammatory response through elevated pro-inflammatory cytokines[43]. Additionally, hyperglycemia weakens the blood-brain barrier, allowing pathogens and inflammatory molecules to penetrate the brain, thereby worsening the infection[44]. It also impairs immune cell function, reducing the body's ability to fight infection and resulting in more severe disease[45]. Furthermore, hyperglycemia increases oxidative stress, leading to CNS cell and tissue damage, which further worsens infection outcomes[46].

Our study has several limitations. First, although headache was identified as a protective factor consistent with previous reports, diagnostic bias may exist—headache could be over-reported in patients with milder disease and under-reported in those with altered consciousness. Second, although this was a prospective multicenter cohort study, all participating centers were tertiary hospitals in Beijing, potentially introducing sample selection bias and limiting the generalizability of our findings. Third, a proportion of cases were clinically diagnosed without definitive microbiological confirmation, which, although reflective of real-world practice, could introduce diagnostic uncertainty. Fourth, sparse data for certain variables may have affected the stability and precision of effect estimates, as sparse data bias is a recognized limitation in observational analyses[47, 48]. Finally, although external validation was conducted, it was performed within the same national context; broader validation across different geographic and healthcare settings is needed to further support the model’s applicability.

From a global perspective, this prognostic model offers a foundational framework that could be adapted and validated in regions with a high burden of CNS infections, such as Southeast Asia, Africa, and South America. Incorporating region-specific factors may further optimize its applicability and assist in improving early clinical decision-making across diverse healthcare settings.

In conclusion, we developed a prognostic model for poor outcomes in community-acquired adult CNS infections. By integrating six routinely available clinical and laboratory indicators, the resulting nomogram offers a practical tool to support early risk stratification and clinical management. While the model demonstrates potential for broader application in regions with high CNS infection burdens, further validation across diverse settings and inclusion of a wider range of pathogens are needed to confirm its generalizability.

## Data Availability

Individual participant data that underlie the results reported in this article, after de-identification (text, tables, figures, and appendices), will be shared. The study protocol, statistical analysis plan, and analytic code will also be available. The data will be available beginning 3 months and ending 5 years following article publication. Access will be granted to researchers who provide a methodologically sound proposal to achieve aims in the approved proposal. Proposals should be directed to zhangylq@sina.com; to gain access, data requestors will need to sign a data access agreement.
